# Effects of a performance and quality improvement intervention on the work environment in HIV-related care: a quasi-experimental evaluation in Zambia

**DOI:** 10.1186/1478-4491-12-73

**Published:** 2014-12-20

**Authors:** Eva Bazant, Supriya Sarkar, Joseph Banda, Webby Kanjipite, Stephanie Reinhardt, Hildah Shasulwe, Joyce Monica Chongo Mulilo, Young Mi Kim

**Affiliations:** Jhpiego/Johns Hopkins University, 1615 Thames Street, Baltimore, MD 21231 USA; Jhpiego Zambia, 8 Ngumbo Road, Long Acres, PO Box 36873, Lusaka, Zambia; Zambia National Service, PO Box 32251, Church road, Lusaka, Zambia

**Keywords:** Delivery of health care, Health facilities/standards, Health personnel, HIV, Prevention of mother-to-child transmission of HIV, Quality improvement, Quality of care, Zambia

## Abstract

**Background:**

Human resource shortages and reforms in HIV-related care make it challenging for frontline health care providers in southern Africa to deliver high-quality services. At health facilities of the Zambian Defence Forces, a performance and quality improvement approach was implemented to improve HIV-related care and was evaluated in 2010/2011. Changes in providers’ work environment and perceived quality of HIV-related care were assessed to complement data of provider performance.

**Methods:**

The intervention involved on-site training, supportive supervision, and action planning focusing on detailed service delivery standards. The quasi-experimental evaluation collected pre- and post-intervention data from eight intervention and comparison facilities matched on defence force branch and baseline client volume. Overall, 101 providers responded to a 24-item questionnaire on the work environment, covering topics of drugs, supplies, and equipment; training, feedback, and supervision; compensation; staffing; safety; fulfilment; and HIV services quality. In bivariate analysis and multivariate analyses, we assessed changes within each study group and between the two groups.

**Results:**

In the bivariate analysis, the intervention group providers reported improvements in the work environment on adequacy of equipment, feeling safe from harm, confidence in clinical skills, and reduced isolation, while the comparison group reported worsening of the work environment on supplies, training, safety, and departmental morale.

In the multivariate analysis, the intervention group’s improvement and the comparison group’s decline were significant on perceived adequacy of drugs, supplies, and equipment; constructive feedback received from supervisor and co-workers; and feeling safe from physical harm (all *P* <0.01, except *P* <0.04 for equipment). Further, the item “provider lacks confidence in some clinical skills” declined in the intervention group but increased in the comparison group (*P* = –0.005). In multivariate analysis, changes in perceived quality of HIV care did not differ between study groups. Provider perceptions were congruent with observations of preparing drugs, supplies, equipment, and in service delivery of prevention of mother-to-child transmission of HIV and antiretroviral therapy follow-up care.

**Conclusions:**

The performance and quality improvement intervention implemented at Zambian Defence Forces’ health facilities was associated with improvements in providers’ perceptions of work environment consistent with the intervention’s focus on commodities, skills acquisition, and receipt of constructive feedback.

## Background

In response to the HIV epidemic, the rapid scale-up of health care services has contributed to crises in human resources across southern African health systems [1, 2]. Zambia’s population of 14 million, of which 12.5% [3] to 14% [4] are infected with HIV, faces considerable challenges in access to health care. The provider-to-population ratio is 0.93 per 1,000, which is far short of the World Health Organization’s recommendation of 2.5 per 1,000 [5]. Growing demand for HIV-related services [6, 7] is likely to put additional stress on human resources [1, 2]. As client loads increase, HIV programmes will need to ensure that quality of care is maintained to standard.

In 2005, the Zambian Defence Force (ZDF), with an HIV prevalence of 28% among personnel, recognized the need to address HIV. The ZDF began offering antiretroviral therapy (ART) and other HIV-related services [8] to its personnel and the wider civilian community.

In Zambia, the military and public sector health systems work together, recognizing that both Ministries serve the Zambian population, and that ZDF Medical Services health facilities in remote settings serve a majority civilian population. The ZDF Medical Services uses Ministry of Health (MOH) tools for provider performance assessment, supervision, health management information system, and logistics management, and the ZDF is involved in development of these tools. To address challenges and further collaboration, for certain services (ART, lab specimens), ZDF Medical Services has signed a memorandum of understanding with the MOH to be able to access commodities from the MOH Medical Stores. MOH supplies drug kits, funds for cleaning materials, and community outreach, and in some cases, MOH staff providers are seconded to ZDF Medical Services.

To improve the quality of HIV-related services, ZDF health facilities began implementing the Standards-Based Management and Recognition® (SBM-R) approach in 2006. In this approach, detailed national standards guide health care providers in performing essential tasks and measuring progress in service delivery [9–12]. SBM-R empowers facility staff to identify and address gaps between current performance and standards through training, supervision, and action planning.

The need to study provider-related aspects of service delivery is increasingly recognized, especially in the face of health policy reforms. In implementation science, important domains of outcomes to study include an intervention’s acceptability and appropriateness (its perceived “fit” in a setting) according to providers, consumers, and other stakeholders, as well as the need to determine how services can be offered at large scale [13]. A positive work environment was found to be associated with health worker rating on quality of care [14]. Policies, standards, and practices that create a work environment in which a functional process of care can be realized have benefits for health outcomes [14, 15].

How providers perceive their work environment, their workloads [16], and the sustainability of services may help predict the success of policy reforms that are expected to increase client volumes for prevention of mother-to-child transmission of HIV (PMTCT) and ART services. As midwives and nurses are increasingly called upon to deliver ART services [17], it will be important to assess their views of their workload and work environment. Provider perspectives can also inform broader health system goals, such as capacity-building and fostering local ownership of services [16, 18–20], as well as shed light on factors that affect the consistent delivery of health services performed to standard [18]. Provider’s views of the quality of care offered to clients is important because providers’ perceived quality of care may be associated with their motivation [21], dedication, productivity, lack of absenteeism, and retention.

We conducted an evaluation study on an SBM-R intervention that was implemented in ZDF facilities to strengthen HIV-related services. Provider performance in ART and PMTCT at these sites [11, 12] has been documented. However, providers’ perspectives on the quality of care have not yet been described. As part of the broader evaluation, this paper’s objective was to determine whether the intervention was associated with improvements in providers’ perceptions on the work environment. This paper also assesses the intervention’s effect on providers’ perceived quality of HIV-related services, and examines this in relation to the observed performance on HIV-related services.

## Methods

### Study design and sample

This study employed a quasi-experimental design with a comparison group and baseline and endline measurements. In 2010, 16 ZDF facilities had already participated in the SBM-R intervention and 38 sites were awaiting their turn in a phased roll-out. The ZDF selected four of these sites to receive the SBM-R intervention in 2010, based on having a high client volume and a need to improve service quality. Four comparison sites were matched to intervention sites on ZDF branch, urban/rural status, and – as closely as possible – service volume and size of catchment population. All three branches of the ZDF were represented in the sample, including two Zambian Air Force facilities, two Zambian National Service facilities, and four Zambian Army facilities. The intervention and comparison sites included camp hospitals in the Army, hospitals in the Air Force, and Camp Clinics in the National Service. Each of these facilities offered, to both military and civilian populations, the services of general medicine, maternal, and child health, including care related to malaria and respiratory infections and HIV-related care. At baseline and endline at least four clinical providers, on average, were working at each facility (Table 1).

**Table 1:** **Provider characteristics in intervention and comparison groups at baseline and endline (n = 101)**

	Baseline			Endline		
	Comparison	Intervention	*P*value	Comparison	Intervention	*P*value
	n = 16	n = 27		n = 29	n = 29	
**Provider cadre** ^**a**^ **(%)**						
Nurse	37.5 (6)	29.6 (8)	0.311	55.2 (16)	20.7 (6)	0.100
Midwife	31.3 (5)	22.2 (6)		13.8 (4)	13.8 (4)	
Clinical officer	31.3 (5)	18.5 (5)		10.3 (3)	17.2 (5)	
Military medical assistant	0.0 (0)	14.8 (4)		13.8 (4)	20.7 (6)	
Pharmacy staff	0.0 (0)	3.7 (1)		0.0 (0)	13.8 (4)	
Laboratory staff	0.0 (0)	0.0 (0)		6.9 (2)	10.3 (3)	
Lay counsellor	0.0 (0)	11.1 (3)		0.0 (0)	3.5 (1)	
**Service areas among clinical staff** ^**b**^	**n = 16**	**n = 23**		**n = 27**	**n = 21**	
PMTCT	68.8 (11)	52.2 (12)	0.313	51.9 (14)	52.4 (11)	0.972
Antenatal care	68.8 (11)	56.4 (13)	0.453	46.2 (12)	52.4 (11)	0.679
ART	56.3 (9)	52.2 (12)	0.808	51.9 (14)	61.9 (13)	0.497
Labour and delivery	56.3 (9)	52.2 (12)	0.808	29.6 (8)	42.9 (9)	0.352
Postnatal care	62.5 (10)	56.5 (13)	0.718	44.4 (12)	42.9 (9)	0.915
Other^c^	0.0 (0)	34.8 (8)	0.007	61.5 (16)	57.1 (12)	0.766
Number of services offered (mean, SD)	3.1 (.42)	3.0 (.40)	0.891	2.9 (.30)	3.1 (.31)	0.724
**Socio-demographics (all providers)**						
Male (%)^b^	66.7	48.2	0.248	58.6	51.7	0.597
Mean age (SD)^d^	35.6 (5.2)	35.1 (8.8)	0.844	35.6 (6.1)	33.8 (7.4)	0.326
Mean years at this health facility (SD)^d^	4.2 (5.1)	6.1 (6.5)	0.316	5.9 (5.5)	5.5 (4.8)	0.750
Mean years ever worked in ZDF (SD)^d^	8.3 (5.4)	11.3 (9.2)	0.246	10.3 (5.7)	9.1 (7.3)	0.522

^a^ *P* value from Fisher’s exact test.

^b^ *P* value from χ^2^ (Provider can work in more than one service).

^c^“Other” service areas include outpatient services, family planning, and administration.

^d^ *P* value from *t*-test.

PMTCT, Prevention of mother-to-child transmission of HIV.

All providers involved with PMTCT and ART services at the time of the assessors’ (interviewers) visit to the facility were invited to participate in the study. Providers agreed to an interview and to be observed during consultations with clients. Clinical providers included nurses, midwives, clinical officers, and military medical assistants – a cadre trained by the Defence School of Health Sciences. Non-clinical providers included laboratory staff, pharmacy staff, and, during the endline round, lay counsellors who were recruited as part of the SBM-R intervention. At baseline, 27 interviews at intervention sites and 16 at comparison sites were conducted, and at endline, 29 at intervention and 29 at comparison sites (total 101 providers). It is not possible to determine whether the same or different providers were interviewed during the two rounds of data collection because of problems assigning unique ID numbers. Participating providers were observed with all clients visiting the facilities on the days of observation, which were 387 clients seeking antenatal care services (191 at baseline and 186 at endline) and 354 clients who were returning for ART follow-up visits (175 at baseline and 179 at endline).

### Description of the intervention

SBM-R is a four-step process that begins by establishing evidence-based performance standards for a service delivery area [9]. These detailed standards describe essential tasks (called verification criteria) and function both as job aids and assessment tools. The ZDF and its partners developed the SBM-R standards for HIV/AIDS-related services used in this study in 2006 and refined them in 2010. The second step in SBM-R is to implement the standards at the facility level. With outside support, a staff team conducts a baseline assessment of services offered at the facility, identifies and analyses performance gaps, and looks for low-cost, local solutions to address them. The third step is to measure the facility’s progress by repeating the performance assessment and to find solutions for remaining performance gaps. The last step is to recognize and reward facilities that, after repeated rounds of assessment and action planning, meet 80% of performance standards.

At the four intervention sites in this study, the SBM-R process began in September 2010 when two staff members from each intervention site attended three days of training on SBM-R. Next, during a three-day site visit, ZDF and Jhpiego staff oriented facility managers and service providers, including all providers who participated in this study, to the quality improvement initiative, national service standards, and SBM-R assessment tools and processes. Providers participated in a 5-day competency-based, onsite training on ART and a 6-day workshop on PMTCT. After these preparations, a team of four to seven staff members at each intervention facility used SBM-R assessment tools to identify strengths and weaknesses in provider performance and support systems, analysed root causes of the problems identified, and developed action plans to address gaps in performance. The action plan was reviewed at supervision visits. The final step in SBM-R, recognition of facilities occurred after endline data collection and hence is not reflected in this analysis.

One emphasis of the intervention was ensuring that providers had the tools to do their jobs; medications, supplies, and equipment for HIV-related services were procured from national stores. Focus was also placed on making timely requests to the national sources. In addition, the following commodities were purchased and provided: key infection prevention supplies and materials, medical equipment, such as blood pressure machines, and office furniture for providers and benches for client waiting areas.

ZDF supervisors drawn from the country’s largest teaching hospital made 2- or 3-day supportive supervision visits to each intervention site twice during the study period for the purpose of the SBM-R intervention. They observed consultations, mentored and coached providers on service delivery to meet the detailed verification criteria of the SBM-R standards, and reviewed and revised the facility’s action plan together with the local team. When staff could not address a problem, the supervisors helped communicate the situation to the leaders at the Defence Forces Medical Services and donors. This SBM-R-specific supervision focused on HIV-related services such as PMTCT and ART initiation and follow-up care. All sites in this evaluation received the usual supervision from the MOH, which pertained to all health areas, including malaria and respiratory infections.

Through the assessment and action planning process at the four intervention facilities, a need for task shifting and staff scheduling emerged. Supervisors helped facilitate the addition of this component to the intervention. PMTCT lay workers were recruited from the local community and trained to take over certain non-clinical responsibilities from service providers such as conducting group education sessions for antenatal clients. In addition, ZDF authorities allowed military medical assistants to perform certain pharmacy and laboratory tasks. SBM-R supervisors also discussed the need to create schedules for facility staff that would allow for more efficient use of human resources.

### Data collection

Baseline data collection occurred in August to October 2010, with assessors staying one to four days in each facility. At six sites, including all of the comparison sites, endline data had to be collected in November and December 2011 (15 months after baseline) prior to an administrative transition and shift in external support. At the two other intervention sites, endline data collection occurred in March and April 2012 (18 to 19 months after baseline) as was originally planned. At baseline and endline, the same provider interview questionnaire was administered.

The assessors were midwives experienced in antenatal care/PMTCT and ART services who worked for the MOH. Two midwives interviewed providers during the baseline round, while four different midwives interviewed providers during the endline round. We trained all of the assessors on the data collection objectives, procedures and tools, the consent process, and ethical issues. ZDF authorities helped the assessors gain admission to the ZDF sites but did not otherwise participate in the study. The assessors also assessed facility readiness for HIV-related service delivery and conducted observations of PMTCT and ART follow-up service consultations using structured observation checklists. These checklists were based on SBM-R assessment tools, which measure service quality as the percentage of essential items present at facilities or the percentage of essential tasks performed by providers. For a detailed description of the methods used to collect the observational data, see the articles published by Kim et al. [11, 12, 22]. Where possible, this paper triangulates the provider interview findings with these previously published observation data.

### Variables and analysis

The 24-item study questionnaire used to measure the work environment was based on the Workplace Climate and Job Satisfaction Survey developed to assess Kenya’s Emergency Hiring Plan [19]. In this tool, the work environment was defined as encompassing the availability of drugs, supplies, and equipment; provider’s receipt of training, feedback, and supervision; compensation; staffing; safety; and fulfilment. Items that were negatively worded were interspersed throughout the list of positively worded items. Providers rated each item with a 5-point Likert scale, with 5 indicating “strongly agree”.

In this same questionnaire, providers were also asked to rate the quality of HIV-related services with one item each for PMTCT, ART facility readiness, ART initiation, ART follow-up services, laboratory, infection prevention, and medical record keeping.

In the provider interview questionnaire, providers’ age, gender, and cadre were recorded, as well as the number of years worked at the facility and years worked in the ZDF. In addition, the tool asks for which HIV-related services and other services were provided, in-service training on various service areas received in the past year, and the number of supervision visits received in the past 6 months.

For greater ability to appreciate the results of the items with a 5-point Likert scale of responses, we analysed the work environment and perceived quality of care items as dichotomous variables. As such, we report the percent of providers who “agreed” or “strongly agreed” (for work environment items) and who rated the service “good” or “very good” in the tables. Separately, the variables were also analysed as continuous measures (on a scale from 1 to 5). Since the findings of items considered continuous were similar to the findings when items were considered binary, we show in the results as binary outcomes.

In bivariate analysis, we used χ^2^ test and Fisher’s exact test for categorical variables. To extend the bivariate analysis, which compared changes over time within each study group (intervention and comparison) in provider ratings of the work environment and perceived service quality, multivariate logistic regression models were estimated. Outcome variables were modelled as a function of intervention status (intervention group and comparison group), time point (baseline and endline), and the interaction of these two variables – controlling for provider cadre (clinical vs. non-clinical) and adjusting for clustering of data within each facility [23]. The interaction term *P* value of the multivariate model assesses whether a change from baseline to endline differed significantly between the intervention and comparison groups. Analysis was performed in Stata 11.0 [24].

### Ethical considerations

The University of Zambia Biomedical Research Ethics Committee and the Johns Hopkins School of Public Health Institutional Review Board approved this study. Providers gave verbal informed consent before participation. Providers were interviewed individually and in private.

## Results

### Provider characteristics

At baseline and endline, on several provider background variables, no significant differences were found between groups (Table 1). Regarding cadre, the intervention group included more non-clinical staff (pharmacy, laboratory, lay counsellor) at both time points, and this difference was more pronounced at endline. Over half of clinical providers in both groups mentioned PMTCT and ART as services they offered at baseline and endline. The two groups did not differ significantly at either time point on provision of services, except for “other” at baseline, which was mentioned by 39% of intervention group providers and 0% of comparison group providers (*P* <0.007). Providers in both groups indicated offering, on average, three services at each time point.

### Training and supervision received

For in-service training in PMTCT in the past year (Table 2), the intervention group increased from 41% at baseline to 80% at endline (*P* = 0.003), whereas the comparison group decreased from 63% to 26% (*P* = 0.022), changes that were significant in the multivariate model (*P* = 0.002). Similar changes for ART training were not significant. The intervention group reported an increase in receiving two or more supervision visits in the past 6 months, from 52% at baseline to 79% at endline (*P* = 0.038). In the comparison group, receipt of supervision was moderately high at both time points (69% and 73%).

**Table 2:** **In-service training and supervision received in intervention and comparison groups**

Item	Intervention group			Comparison group			Adjusted model – interaction *P*value^b^
	Baseline, n = 27	Endline, n = 29	*P*value^a^	Baseline, n = 16	Endline, n = 29	*P*value^a^	
**Percentage (%) who received in-service training in past year on:**							
Prevention of mother-to-child transmission of HIV	40.7 (11)	79.3 (23)	0.003	62.5 (10)	25.8 (8)	0.022	0.002
Antiretroviral therapy	55.6 (15)	75.9 (22)	0.109	62.5 (10)	44.8 (13)	0.266	0.218
Sexually transmitted infections	59.3 (16)	58.6 (17)	0.961	56.3 (9)	37.9 (11)	0.236	0.455
Tuberculosis	33.3 (9)	34.5 (10)	0.928	37.5 (6)	25.8 (7)	0.355	0.456
Infection prevention	37.0 (10)	55.2 (16)	0.174	50.0 (8)	58.6 (17)	0.588	0.365
Percentage (%) who received 2+ supervision visits in the past 6 months	52.2 (12)	79.3 (23)	0.038	69.2 (13)	73.1 (26)	0.808	0.055

^a^ *P* value from χ^2^ at baseline or at endline.

^b^Interaction term *P* value from multivariate linear regression models of each result on the intervention status, time point, and interaction of these two variables, while controlling for provider cadre and ZDF branch, and accounting for clustering of responses within each facility.

### Work environment

In the bivariate analysis, in the intervention group, a statistically significant improvement occurred in five perceived work environment items, including an improvement in providers’ views on adequacy of equipment and providers feeling safe from physical harm when working in the facility (Table 3). There was a statistically significant reduction in providers feeling isolated, providers lacking confidence in clinical skills, and providers feeling overworked (Table 4). In the comparison group, a statistically significant decline occurred in five work environment items, including adequacy of supplies, training offered in critical skills, training offered of interest to providers, providers feeling safe from physical harm, and the department having a good morale.

**Table 3:** **Percent of providers who agreed with positive statements on the work environment, by study group** ^**a**^

Items	Intervention			Comparison			Adjusted model – interaction *P*value^b^
	Baseline, n = 27	Endline, n = 29	*P*value^b^	Baseline, n = 16	Endline, n = 29	*P*value^b^	
**Drugs, supplies, and equipment**							
Drugs are adequate	70.3	82.8	0.28	87.5	72.4	0.25	0.006
Supplies are adequate	74.1	82.8	0.44	100.0	65.5	0.01	0.001
Equipment (e.g., blood pressure cuffs) is adequate	61.5	89.7	0.01	68.8	48.3	0.19	0.036
**Training, feedback, and supervision**							
Job expectations are known	96.3	100.0	0.30	87.5	89.2	0.86	–
Constructive feedback received from supervisor	73.1	89.7	0.11	93.8	75.0	0.13	0.02
Constructive feedback received from co-worker	69.2	86.2	0.13	93.8	82.8	0.31	0.01
Provider received recognition, either as individual or as part of the team	84.6	68.9	0.18	93.8	90.1	0.65	0.76
Training is provided in critical skills	81.5	82.8	0.90	81.3	44.8	0.02	0.004
Training provided is of interest to provider	66.7	65.5	0.93	87.5	44.8	0.01	0.004
**Compensation/salary**							
Provider received appropriate salary	76.9	65.5	0.36	75.0	55.2	0.20	0.750
Provider received timely salary	96.0	96.6	0.92	100.0	100.0	–	–
Provider received appropriate leave time	72.0	58.6	0.31	73.3	55.1	0.25	0.701
**Staffing and safety**							
Number of provider staff is adequate	37.0	34.5	0.85	63.0	50.0	0.43	0.906
Number of support staff is adequate	62.9	62.0	0.95	62.5	55.2	0.64	0.632
Provider feels safe from physical harm when working in the facility	77.8	96.6	0.03	100.0	82.7	0.08	0.001
**Fulfilment**							
Provider feels job is fulfilling	92.5	89.7	0.70	93.8	86.2	0.45	0.813
Department has good morale	81.5	75.9	0.61	87.5	58.6	0.05	0.459
Provider feels work is important	100.0	96.3	0.36	100.0	100.0	----	----
Provider feels work is valued by the community	100.0	92.9	0.16	93.8	96.6	0.67	0.001
Your work has a positive impact on the health of the community	100.0	96.6	0.34	100.0	100.0	----	----

^a^Response scale was 1 to 5, with 5 meaning “strongly agree” and 1 meaning “strongly disagree”. This table reflects the percent of respondents who “agreed” and “strongly agreed”.

^b^Bivariate results show *P* value from *t*-test comparing values at baseline or at endline. Interaction term *P* value is from multivariate logistic regression models of each result on the intervention status, time point, and interaction of these two, while controlling for provider cadre and ZDF branch, and accounting for clustering of responses within each facility.

**Table 4:** **Percent of providers who agreed with negative statements on the work environment, by study group** ^**a**^

Item	Intervention			Comparison			Adjusted model – interaction *P*value
	Baseline, n = 27	Endline, n = 33	*P*value^b^	Baseline, n = 16	Endline, n = 29	*P*value^b^	
Provider feels isolated	25.9	3.4	0.02	6.3	6.9	0.94	0.232
Provider lacks confidence in some clinical skills	44.4	13.8	0.01	25.0	42.8	0.25	0.005
Provider feels overworked	59.3	34.5	0.07	43.8	62.1	0.25	0.135
Provider feels job is stressful	63.0	48.3	0.28	75.0	75.9	0.95	0.507

^a^Response scale is 1 to 5, with 5 meaning ”strongly agree” and 1 meaning “strongly disagree”. This table reflect the percent of respondents who ‘agreed’ and ‘strongly agreed’.

^b^Bivariate results show *P* value from *t*-test comparing values at baseline or at endline. Interaction term *P* value is from multivariate logistic regression models of each result on the intervention status, time point, and interaction of these two, while controlling for provider cadre and ZDF branch, and accounting for clustering of responses within each facility.

In the multivariate analysis, the intervention group’s improvement and the comparison group’s decline were statistically significant for three items on drugs, supplies, and equipment (*P* = 0.006, *P* = 0.001, and *P* = 0.036, respectively; Table 3). This pattern was also apparent for constructive feedback received from supervisor and from co-workers (*P* = 0.02 and *P* = 0.01, respectively) and on feeling safe from physical harm when in the facility (*P* = 0.004 and *P* = 0.001, respectively). For the items on training (in critical skills and of interest to provider), this was significant in the multivariate analysis (*P* = 0.004) as it fell dramatically in the comparison sites but remained at relatively high levels at intervention sites. The negatively worded item, “provider lacks confidence in some clinical skills” (Table 4) declined in intervention group and increased in comparison group (*P* = –0.005).

### Perceived quality of care

In the bivariate analysis, in the intervention group, a statistically significant improvement occurred in providers’ perceptions of five service areas (Table 5). These were ART treatment readiness, ART initiation, ART follow-up, PMTCT, and laboratory. An improvement in providers’ perception of three services was also found in the control group for ART treatment readiness, ART initiation, and laboratory, in the bivariate analysis. In the multivariate analysis, none of the changes within groups on providers’ perceptions of quality of services were statistically significantly different.

**Table 5:** **Percent of providers who rated quality as good in several HIV/AIDS service areas** ^**a**^

HIV/AIDS service area	Intervention group			Comparison group			Adjusted model – interaction *P*value^c^
	Baseline, n = 27	Endline, n = 29	*P*value^b^	Baseline, n = 16	Endline, n = 29	*P*value^b^	
ART treatment readiness	80.0	100.0	0.01	60.0	91.3	0.02	–^d^
ART initiation	72.0	92.6	0.05	61.5	96.2	0.004	0.463
ART follow-up	68.0	96.6	0.005	73.3	76.9	0.80	0.121
PMTCT	80.0	100.0	0.01	57.1	81.5	0.10	–^d^
Laboratory	48.0	79.3	0.02	7.1	50.0	0.007	0.402
Infection prevention	92.0	89.7	0.77	73.3	82.1	0.50	0.169
Medical recording keeping	84.0	93.1	0.29	73.3	85.2	0.35	0.921

^a^Response scale was 1 to 5, with 5 meaning “strongly agree” and 1 meaning “strongly disagree”. This table reflects the percent of respondents who “agreed” and “strongly agreed”.

^b^ *P* value from *t*-test at baseline or at endline.

^c^Interaction term *P* value from multivariate logistic regression models of each result on intervention status, time point, and interaction of these two variables, while controlling for provider cadre and ZDF branch and accounting for clustering of responses within each facility.

^d^The dash means that the Odds Ratio coefficient for the interaction term = 1, thus no *P* value is associated with it.

ART, Antiretroviral therapy; PMTCT, Prevention of mother-to-child transmission of HIV.

### Comparing interview and observational data

**Figure 1:**
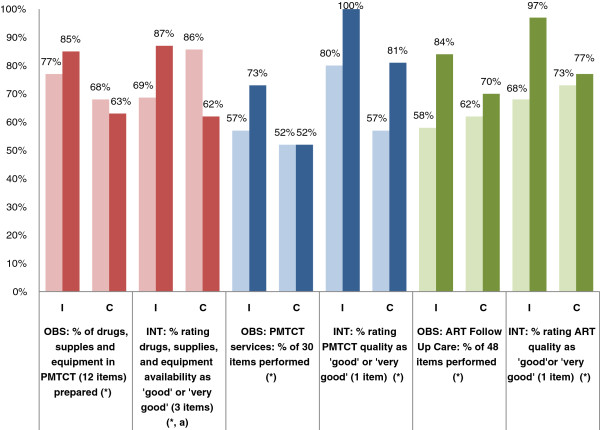
**Results of observations of performance and provider interviews on readiness and PMTCT/ART Services in ZDFHS.** I, Intervention; C, Comparison; OBS, Observations (n = 387); INT, Interviews (n = 101). *P <0.05 in bivariate analysis. (a) *P* <0.05 in multivariate analysis. Lighter color, Baseline; Darker color, Endline.

In Figure 1, results from third-party observations of provider performance and providers’ perceptions of quality of specific services in interviews are shown side-by-side for comparison. Regarding the availability of drugs, supplies, and equipment, providers’ ratings are congruent with observations of drugs, supplies, and equipment availability in PMTCT. At intervention sites, providers’ perceptions that the quality of PMTCT services and ART follow-up care improved are also consistent with observations of consultations. However, at comparison sites, providers reported an improvement in PMTCT service quality that was not observed.

## Discussion

This paper contributes to the evidence base supporting a standards-based performance and quality improvement approach as an effective intervention in settings facing severe shortages in human resources for health. Because providers are key actors in any policy reform that affects service delivery and use [25], their work-related attitudes and impressions can have an enormous impact on the success of new initiatives and also help inform strategies for managing increased client loads. For example, providers’ negative attitudes toward ART patients and defaulters have been found to be a barrier to clients’ ART adherence [26]. Staffing levels and provider stress may act as barriers or facilitating factors in strengthening PMTCT and ART services [27]. In the context of HIV-related care in Zambia, our intervention addressed many of the factors that directly affect providers’ work environment and performance [18] and was associated with improvements in both areas [12, 22].

### Implications of results

#### In-service training and supervision

There was a striking difference between groups in PMTCT training received in the last year, with intervention providers reporting a sharp increase and comparison providers reporting a sharp decline by endline. The gain at intervention sites is a direct result of the SBM-R intervention, which included special training courses on PMTCT for both providers and lay counsellors. Comparison sites reported relatively high levels of training in the last year, which may have been due to another partner organization’s work in supporting and strengthening these sites before the start of this study, especially in logistics [28]. Similarly, the comparison group reported moderately high levels of supervision received in the last 6 months, which may be explained by the fact that supervision is routinely offered at ZDF sites by both the ZDF and MOH. MOH supervision covered many health areas while the SBM-R supportive supervision was focused on HIV-related care, and mentoring providers in the details of service delivery according to the standards. The SBM-R supervision also focused on addressing gaps and action planning. In general, supervision may be perceived, and likely conducted, differently in different contexts. In a study in Kenya and Benin, routine supervision was sometimes perceived by providers as an exercise in control, in which attention was focused on mistakes [29]. In the same study, feeling neglected by supervisors or the health system was demotivating to providers; the desired supervision style, as described by providers, is supportive, instructive, needs-oriented, and participatory, including the provision of timely, constructive feedback. In the present study in Zambia, supervisors’ constructive feedback and providers’ confidence in their own clinical skills increased significantly at intervention sites only. This finding likely reflects SBM-R’s emphasis on supportive supervision.

#### Aspects of the work environment

Providers’ perspectives at intervention sites suggested significant improvements in several aspects of the work environment. Notably, ratings for the adequacy of drugs, supplies, and equipment all increased, consistent with the emphases of the intervention. The availability of such commodities is critical for provider motivation and their sense being able to conduct their work as professionals [29, 30]. In addition, in the intervention group, there was an overall decrease in feeling “isolated” and an increase in level of confidence in skills by endline, relative to the comparison group, changes that may be due to SBM-R’s focus on supportive supervision and teamwork approach to problem-solving. An improvement in providers’ feeling safe from physical harm in the facility may be due to the intervention’s focus on infection prevention and control, and supplies for this. Comparison sites reported declines in several aspects of the work environment. Items such as “provider feels work is valued by the community”, however, were high in both groups at baseline and endline. This may be due to clients’ general appreciation of access to HIV-related care, especially in remote settings. The military health system in Zambia offers health services not only to its members, but also to the civilian community.

#### Service quality

Improvements in the intervention groups’ perceived quality of ART follow-up and PMTCT services were corroborated by observations, whereas, in the comparison group, provider reports of PMTCT quality were not always consistent with the observational findings. This suggests that providers who were part of the SBMR process may have a more accurate view of quality of services. However, despite the intervention group’s reports of quality improvements in five of seven HIV-related services versus three of seven for the comparison group, none of these changes in perceptions were significant in multivariate analysis. Nevertheless, the SBM-R approach, which includes provider training, action planning, and supportive supervision, may help address operational and human resource challenges posed by the rapid scale-up of PMTCT in Zambia, resulting from the recommended Option B+ approach [6].

### Limitations

This study had several limitations. First, provider interview data is self-reported and may be subject to personal bias. Working at an intervention site also may have affected providers’ responses; because providers are active participants in the SBM-R process, they may have been disposed to rate services more highly during the endline round. However, we did triangulate provider interviews and observations where possible to check for congruency. Second, the small sample size of interviews conducted in each group at each time point may have limited our ability to detect differences in multivariate analyses. Third, we only have data on providers’ perspectives; clients were not interviewed so we lack a full picture of service quality and the work environment. Regarding generalizability, the eight sites may not be representative of all ZDF facilities, and differences may exist between ZDF and MOH facilities. However, we did include all three branches of the ZDF in our study. Further, we did address many of the working conditions and aspects of performance considered important in other low-resource settings [19], as well as in studies conducted at the Zambian MOH facilities [30] and hospitals [31].

### Recommendations for future studies

In the future, longitudinal studies can investigate whether the interventions evaluated in this study are associated with provider retention, continued motivation and productivity, maintenance of gains in performance/quality of care, and sustainability of services [16, 20]. It would also be valuable to triangulate providers’ views on the work environment with supervisors’ and clients’ perspectives. Future studies can also examine the specific effects of the fourth stage of SBM-R, the “R”, for external, public recognition of the facility, as well as triangulate provider views on quality of services with client outcomes such as adherence to ART and retention in care. Client provider ratios at facilities should be assessed before and during an intervention to help understand the context of the care provided.

## Conclusions

To meet the demands of increasing client loads in Zambia, many more providers will need to be trained and supported through supervision, as well as have access to adequate drugs, supplies, and equipment. The SBM-R/task-shifting intervention evaluated in this study shows promise in helping to address such needs in a setting facing severe shortages in human resources for health. The performance and quality improvement intervention implemented at ZDF health facilities was associated with improvements in providers’ perceptions of work environment consistent with the intervention’s focus on commodities, skills, feedback, and supervision. The changes in providers’ perceptions of work environment at the comparison sites suggest that there were other influences at those sites.

Health systems in many low-resource settings are paying increasing attention to making employees feel valued and supported, and a supervisor’s attention plays a key role in this effort [19]. How best to reward and motivate providers in a sustainable manner is an ongoing question for policymakers in the context of service decentralization and scale-up. Multi-dimensional supervision with whole-site facility assessments, which characterizes the SBM-R approach, has the potential to motivate providers by highlighting the benefits of assessment to providers’ knowledge, skills, teamwork, and outcomes for clients; by offering providers training and support of an experienced off-site mentor and facilitator; and by having on-site managers participate in assessment and recognize providers for their contributions [32]. As Shelton noted, “*as we design and implement programs, we need to be mindful of the perspectives of providers on the frontline*” [16].

## Abbreviations

ART: Antiretroviral therapy
MOH: Ministry of Health
PMTCT: Prevention of mother-to-child transmission
SBM-R: Standards-based management and recognition
ZDF: Zambian defence force.
